# Role of phosphorylated extracellular signal-regulated kinase, calcitonin gene-related peptide and cyclooxygenase-2 in experimental rat models of migraine

**DOI:** 10.3892/mmr.2015.3616

**Published:** 2015-04-15

**Authors:** XIAOMENG DONG, YAOZHI HU, LONG JING, JINBO CHEN

**Affiliations:** 1 Binzhou Medical University, Yantai, Shandong 264003, P.R. China; 2 Department of Neurology, The Affiliated Hospital of Binzhou Medical University, Binzhou, Shandong 256603, P.R. China; 3 Department of Special Inspection, Binzhou People’s Hospital, Binzhou, Shandong 256610, P.R. China

**Keywords:** migraine, phosphorylated extracellular signal-regulated kinase, calcitonin gene related peptide, cyclooxygenase-2, nitroglycerin-induced migraine model, electrical stimulation of the trigeminal ganglion model

## Abstract

Although migraine is a common neurological condition, the pathomechanism is not yet fully understood. Activation of the trigeminovascular system (TVS) has an important function in this disorder and neurogenic inflammation and central sensitization are important mechanisms underlying this condition. Nitroglycerin (NTG) infusion in rats closely mimics a universally accepted human model of migraine. Electrical stimulation of the trigeminal ganglion (ESTG) of rats can also activate TVS during a migraine attack. Numerous studies have revealed that phosphorylated extracellular signal-regulated kinase (p-ERK), calcitonin gene-related peptide (CGRP) and cyclooxygenase-2 (COX-2) are involved in pain and nociceptive pathways. However, few studies have examined whether p-ERK, CGRP and COX-2 are involved in neurogenic inflammation and central sensitization. In the present study, the expression of p-ERK, CGRP and COX-2 was detected in the dura mater, trigeminal ganglion (TG) and spinal trigeminal nucleus caudalis in NTG-induced rats and ESTG models by immunohistochemistry. The three areas considered were crucial components of the TVS. The selective COX-2 inhibitor nimesulide was used in ESTG rats to examine the association between p-ERK, CGRP and COX-2. The results demonstrated that p-ERK, CGRP and COX-2 mediated neurogenic inflammation and central sensitization in migraine. In addition, the expression of p-ERK and CGRP was attenuated by the COX-2 inhibitor.

## Introduction

Migraine is a complex neurovascular disorder that is often manifested as severe, episodic and predominantly unilateral throbbing head pain with hypersensitivity to light, sound and movement (1–3), affecting ~15% of the adult population worldwide, and can lead to ischemic stroke, depression, cognitive impairment and epilepsy (3). However, the mechanisms underlying migraine are not completely understood. The trigeminovascular system (TVS) mediates neurogenic inflammation, which is characterized by meningeal vascular expansion, plasma protein leakage and mast cell degranulation (4).

Another important symptom, which is often observed in patients suffering from chronic migraine, is cutaneous allodynia (5). Cutaneous allodynia is believed to be a result of central sensitization, which is also mediated by the TVS (6,7). The neurons in the spinal trigeminal nucleus caudalis (TNC) receive input signals from the dura mater and periorbital skin (2). As a result of this sensitization, non-noxious stimuli of the skin are perceived as painful (5). However, limited information is available on the tissue factors that participate in central sensitization and the mechanisms that maintain the activation of meningeal nociceptors that cause neurogenic inflammation and sensitization.

Extracellular signal-regulated protein kinases (ERK) are mitogen-activated protein kinases that are activated by membrane depolarization and calcium influx (8), and known to be one of the intracellular signaling pathways involved in neuronal plasticity (9,10). The phosphorylation of ERK (p-ERK) is a response to the noxious stimulation of peripheral transient receptor potential vanilloid receptor 1 (TRPV1) (11). The noxious information is carried to the peripheral nerve endings and the TNC.

Calcitonin gene-related peptide (CGRP) is a key neuropeptide in the pathophysiology of migraine and the levels of plasma CGRP are increased in the external jugular during attacks in migraine patients (12,13). The stimulation of the trigeminal ganglion (TG) in animal models of migraine causes the release of neuropeptides, including CGRP, substance P and neurokinin A, which induces a series of peripheral and central events, including vasodilatation (14), inflammation and neuronal activation (15,16).

In addition to CGRP, cyclooxygenase-2 (COX-2) is an important peripheral mediator of inflammation and pain. COX-2 can increase prostaglandin E_2_ (PGE_2_) production in the central nervous system and contribute to the severity of pain responses in inflammatory pain (17,18). Nonsteroidal anti-inflammatory drugs and selective inhibitors of COX-2 (e.g. nimesulide; NM) have been used in migraine therapy for decades, and can reduce plasma protein extravasation in experimentally induced neurogenic inflammation of the rat dura mater (19). This drug can also attenuate c-Fos expression in the TNC of the electrical stimulation of the trigeminal ganglion (ESTG) model (13).

Systemic administration of nitroglycerin (NTG), a nitric oxide donor, can trigger a spontaneous-like migraine attack in migraineurs, however, not in healthy individuals (20). In rats, subcutaneous administration of NTG (10 mg/kg) can mimic a human migraine attack, which is the closest possible simulation of the human NTG model (21,22). Unilateral electrical stimulation of the trigeminal ganglion of rats (UESTG) can induce structural alterations in CGRP positive sensory nerve terminals and cause plasma protein leakage in the dura mater (4). Therefore, UESTG can induce chemical and vascular alterations that are similar to those observed during a migraine attack.

The dura mater, TG and TNC are key parts of the TVS and are essential in the process of inflammation and sensitization in migraine (23). CGRP, p-ERK and COX-2 are strongly associated with pain, particularly with the transmission of nociceptive information. However, the function of these three substances in neurogenic inflammation, central sensitization and the intrinsic link among them during migraine attacks has not been thoroughly examined.

The aim of the present study was to determine whether p-ERK, CGRP and COX-2 are involved in migraine neurogenic inflammation and central sensitization in the NTG-induced migraine rat model. UESTG migraine model and NM were used to further assess the possible functional connections between p-ERK, CGRP and COX-2 in migraine. Immunohistochemistry (IHC) was used to analyze the protein expression of p-ERK, CGRP and COX-2.

## Materials and methods

### Animals

In total, 60 male Sprague-Dawley rats weighing 280–320 g (Vital River Laboratory Animal Technology Co., Ltd., Beijing, China) were used. All rats were kept under standard laboratory housing conditions with a 12 h light-dark cycle and had free access to food and water. All experimental protocols were approved by the Ethics Committee for the Use of Experimental Animals at Binzhou Medical University (Binzhou, China). All procedures were undertaken with utmost caution to minimize the suffering of animals. All rats were randomly divided into four groups: Blank (n=6), NTG (n=36), ESTG (n=18) and NM (n=6) groups. The NTG group (n=36) was then randomly divided into the NTG model (n=18) and vehicle-treated (NS; n=18) groups. The ESTG group (n=18) was also randomly divided into the ESTG model (n=6) and sham-operation (SO; n=6) groups.

### Experimental protocols

#### Administration of drugs

The rats in the NTG model group received weekly subcutaneous (s.c.) injections of NTG (Beijing Yimin Pharmaceutical Co., Ltd., Beijing, China) at a dose of 10 mg/kg for five continuous weeks. For the control, the vehicle solution (0.9% NaCl) was administered weekly via s.c. injections to the NS group rats for five continuous weeks.

The rats in the NM group received intragastric administration of the selective COX-2 inhibitor NM (Hainan Zhongrui Kangzhi Pharmaceutical Co., Ltd., Hainan, China), which was dissolved in saline in a volume of 10 ml/kg at a dose of 6 mg/kg/day for 7 days. Subsequently, 30 min after the last drug administration, the rats were anesthetized with 10% chloral hydrate (4 ml/kg; i.p.) and subjected to UESTG.

#### ESTG

Rats in the ESTG model group were anesthetized with 10% chloral hydrate (4 ml/kg, i.p.) and placed in a stereotaxic frame (ZH-B; Zhenghua Biological Instrument Co., Ltd., Huaibei, China). The calvarium was exposed by a midline incision. A hole was drilled with a cranial drill 3.2–3.4 mm posterior to and 2.8–3.2 mm laterally from the bregma. A disposable concentric needle electrode (DCN37; Alpine Biomed Corp., Fountain Valley, CA, USA) was lowered into the right TG (at a depth of ~9.2 mm from the dura mater). TG was electrically stimulated for 30 min with square pulses at 10 Hz and 0.5 mA with a pulse duration of 5 ms. Correct electrode placement of the electrode needle was confirmed by ipsilateral contraction of the masseter muscle during stimulation.

The rats in the SO group (n=6) underwent a surgical procedure similar to that performed in the rats of the ESTG group. However, the concentric bipolar electrode was only lowered into the right TG and was maintained for only 30 min. The TG was not electrically stimulated.

All rats in the NTG model and NS groups were anesthetized with chloral hydrate (4 ml/kg, i.p.) at 30 min, 1 or 3 h after NTG or NS administration (n=6 each). The rats in the ESTG model and SO groups were anesthetized for 30 min after stimulation or sham-stimulation. Following being anesthetized, all rats were transcardially perfused with 100–200 ml of 0.1 M phosphate-buffered saline (PBS; pH 7.4; ZSGB-BIO, Beijing, China), followed by 500 ml of cold and freshly made 4% paraformaldehyde (Tianjin No. 1 Organic Chemical Plant, Tianjin,China) in 0.1 M PBS. Portions of the cervical spinal cords, representing the lowest part of the TNC, between 5 and 11 mm caudal to the obex were removed and postfixed overnight for IHC. The ipsilateral dura mater and TG of the rats in the ES model, SO and NM groups were also dissected and prepared for IHC.

#### IHC

The dura mater, TG and TNC were fixed in 4% formalin for at least 24 h, washed with 0.9% saline and processed with ethanol and xylene solutions. The preparations were then embedded in paraffin, cut into 4-*µ*m thick sections and mounted on glass slides following conventional procedures. The sections were rinsed in PBS for 15 min and boiled in citrate buffer (pH 6.0) for 15 min for antigen retrieval. Following boiling, the sections were immersed in methanol containing 0.3% H_2_O_2_ for 20 min. Sections were blocked with 1% bovine serum albumin at 21°C for 10 min prior to incubation overnight at 4°C with one of the following antibodies: Goat polyclonal anti-CGRP (1:300; cat. no. ab36001; Abcam, Cambridge, MA, USA), rabbit polyclonal anti-COX-2 (1/350; cat. no. ab15191; Abcam) or mouse monoclonal anti-p-ERK (1/50; cat. no. sc-7383; Santa Cruz Biotechnology, Inc., Santa Cruz, CA, USA). Following overnight incubation, preparations were washed with PBS and incubated with MaxVision™ kits (Fuzhou Maixin Biotech. Co., Ltd., Fuzhou, China), including monoclonal rabbit anti-goat (Kit-5107), polyclonal goat anti-rabbit (Kit-5004) and monoclonal goat anti-mouse (Kit-5001) immunoglobulin G secondary antibodies, at 37°C for 30 min. Following incubation, the preparations were washed thoroughly, incubated in 3,3′-diaminobenzidine tetrahydrochloride solution for color detection and counterstained with hematoxylin.

#### Image acquisition and statistical analysis

The immunolabeled specimens were examined under an Olympus BX51 microscope (Olympus, Tokyo, Japan) equipped with a DP72 camera (Olympus). Five images of each slide covered with cultured cells were captured under x40 fixed magnification for the TG and TNC and x100 for the dura mater. The measurement parameter was the mean optical density (MOD) calculated using Image-Pro Plus 6.0 software (Media Cybernetics, Silver Spring, MD, USA).

All values are presented as the mean ± standard deviation. Independent Student’s t-test was used to compare data from two groups. One-way analysis of variance followed by Tukey’s post-hoc test was applied when more than two groups of data were compared. P<0.05 was considered to indicate a statistically significant difference. GraphPad Prism 5.0 software (GraphPad Prism Software Inc., San Diego, CA, USA) was used for statistical analysis.

## Results

### Effect of NTG infusion on p-ERK, CGRP and COX-2 protein expression in the dura mater, TG and TNC

**Figure 1:**
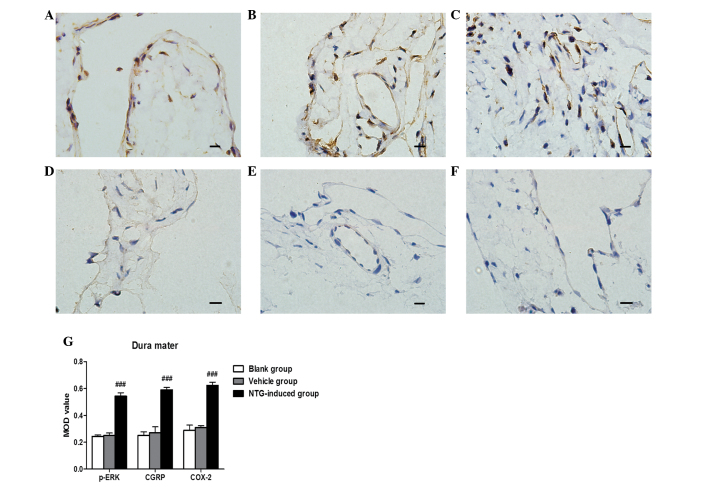
Representative images of p-ERK, CGRP and COX-2 immunoreactivity in the dura mater 30 min after (A–C) NTG or (D–F) vehicle infusion by immunohistochemistry and (G) analysis of the MOD of p-ERK, CGRP and COX-2 expression in the dura mater. An increase in (A) p-ERK, (B) CGRP and (C) COX-2 expression was observed in the dura mater following NTG infusion and the MOD value for NTG-treated rats was significantly higher than in the vehicle and blank groups. (^###^P<0.001, compared with the vehicle and blank groups; n=6 in each group; error bars indicate standard deviation; scale bar=100 *µ*m). MOD, mean optical density; p-ERK, phosphorylated extracellular signal-regulated kinase; CGRP, calcitonin gene-related peptide; COX-2, cyclooxygenase-2; NTG, nitroglycerin.

**Figure 2:**
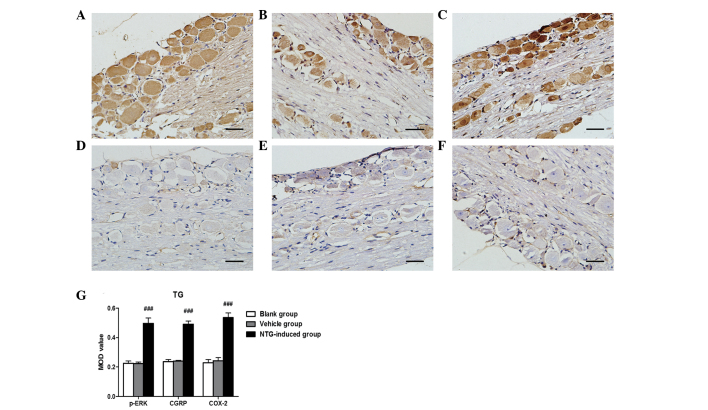
Representative images of p-ERK, CGRP and COX-2 immunoreactivity in the TG 30 min after (A–C) NTG or (D–F) vehicle infusion by immunohistochemistry and (G) analysis of the MOD of p-ERK, CGRP and COX-2 expression in the TG. An increase in (A) p-ERK, (B) CGRP and (C) COX-2 expression was observed in the TG following NTG infusion and the MOD value for NTG-treated rats was significantly higher than in the vehicle and blank groups. (^###^P<0.001, compared with the vehicle and blank groups; n=6 in each group; error bars indicate standard deviation; scale bar=100 µm). MOD, mean optical density; p-ERK, phosphorylated extracellular signal-regulated kinase; CGRP, calcitonin gene-related peptide; COX-2, cyclooxygenase-2; TG, trigeminal ganglion; NTG, nitroglycerin.

**Figure 3:**
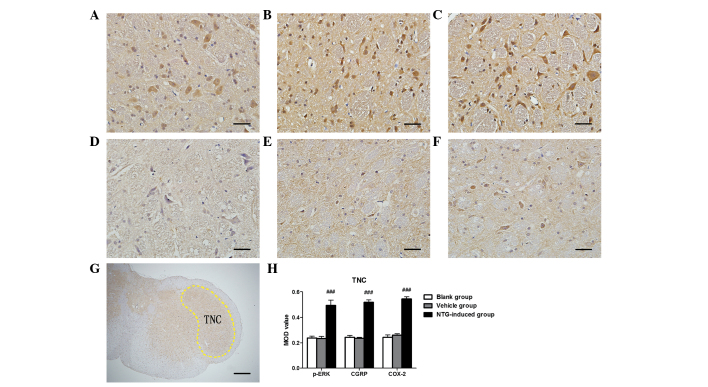
Representative images of p-ERK, CGRP and COX-2 immunoreactivity in the TNC 30 min after (A–C) NTG or (D–F) vehicle infusion by immunohistochemistry, (G) rat TNC section nuclei and (H) analysis of the MOD of p-ERK, CGRP and COX-2 expression in the TG. An increase in (A) p-ERK, (B) CGRP and (C) COX-2 expression was observed in the TNC following NTG infusion and the MOD value for NTG-treated rats was significantly higher than in the vehicle and blank groups. [^###^P<0.001, compared with the vehicle and blank groups; n=6 in each group; error bars indicate standard deviation; scale bar (A–F)=100 *µ*m; scale bar (G)=1 mm]. MOD, mean optical density; p-ERK, phosphorylated extracellular signal-regulated kinase; CGRP, calcitonin gene-related peptide; COX-2, cyclooxygenase-2; TNC, trigeminal nucleus caudalis; NTG, nitroglycerin.

**Figure 4:**
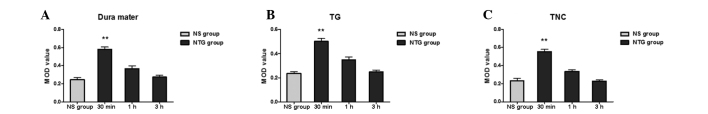
Expression of p-ERK in the (A) dura mater, (B) TG and (C) TNC following NTG infusion. As shown in the histogram, the phosphorylation of ERK following NTG infusion of the dura mater, TG and TNC demonstrated a temporal profile. Significantly higher levels of p-ERK were found in the dura mater, TG and TNC 30 min after NTG administration compared with the controls. The p-ERK levels gradually decreased and were close to the basal level by 3 h. (^**^P<0.01, compared with the NS group; n=6 in each group; error bars indicate standard deviation). p-ERK, phosphorylated extracellular signal-regulated kinase; TNC, trigeminal nucleus caudalis; TG, trigeminal ganglion; NTG, nitroglycerin; NS, vehicle-treated rats.

Based on IHC analysis, CGRP and COX-2 protein were strongly expressed in the dura mater, TG and TNC 30 min, 1 or 3 h after NTG infusion compared with the control rats (P<0.001; Figs. 1–3). No significant difference between CGRP and COX-2 expression was observed 30 min, 1 or 3 h after NTG infusion. For p-ERK, a temporal profile of NTG-induced phosphorylation in the TVS was observed. Significantly higher levels of p-ERK were found in the dura mater (F=72.72; P<0.01), TG (F=68.08; P<0.01) and TNC (F=128.3; P<0.01) 30 min after NTG administration compared with the controls. The p-ERK levels gradually decreased and were close to the basal level by 3 h (Fig. 4). Vehicle (NS)-treated rats demonstrated low basal levels of p-ERK, CGRP and COX-2 protein expression in the dura mater, TG and TNC at 30 min, 1 and 3 h after vehicle infusion (Figs. 1–3). Furthermore, no significant difference in the expression of p-ERK, CGRP and COX-2 was observed at 30 min, 1 or 3 h in vehicle (NS)-treated rats.

### Effect of electrical stimulation and pretreatment with NM on p-ERK, CGRP and COX-2 protein expression in the dura mater, TG and TNC

**Figure 5:**
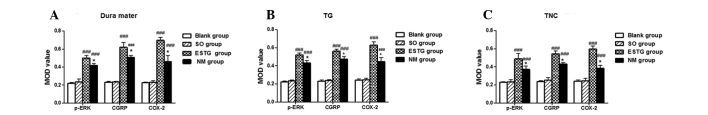
Effect of electrical stimulation and pretreatment with NM on the protein expression of p-ERK, CGRP and COX-2 in the (A) dura mater, (B) TG and (C) TNC. As shown in the histogram, following electrical stimulation, a significant increase in p-ERK, CGRP and COX-2 was observed in the TNC, ipsilateral dura mater and TG compared with the sham-surgery and blank groups. Pretreatment with NM demonstrated a significant decrease in p-ERK, CGRP and COX-2 MOD values in the TNC, ipsilateral dura mater and TG compared with the electrically-stimulated rats, however this was higher than in the sham-surgery and blank groups. No differences were detected between the sham-surgery and the blank groups. (^*^P<0.05, compared with the electrically-stimulated group; ^###^P<0.001, compared with the sham-surgery and blank groups; n=6 in each group; error bars indicate standard deviation). MOD, mean optical density; p-ERK, phosphorylated extracellular signal-regulated kinase; CGRP, calcitonin gene-related peptide; COX-2, cyclooxygenase-2; NM, nimesulide; ESTG, electrical stimulation of the trigeminal ganglion; SO, sham operation; TNC, trigeminal nucleus caudalis; TG, trigeminal ganglion.

**Figure 6:**
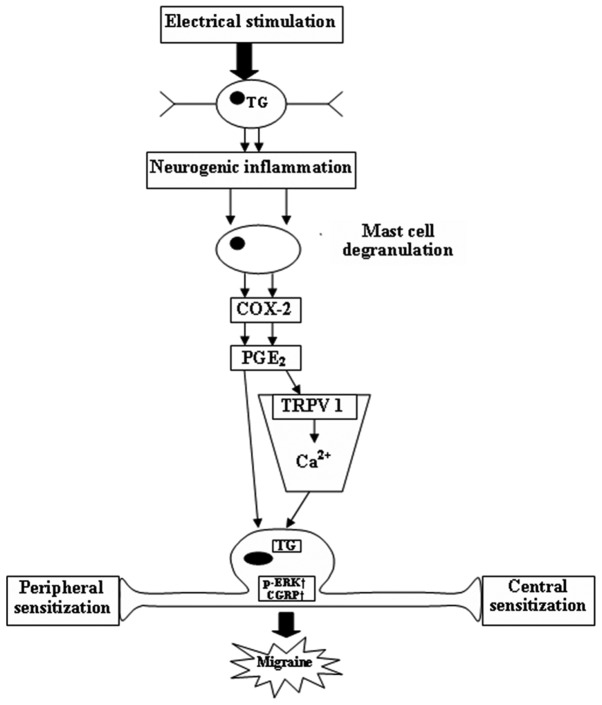
Connections between p-ERK, CGRP and COX-2 in the pathophysi-ological mechanisms of migraine. Electrical stimulation of the TG leads to neurogenic inflammation in trigeminal neurons and glial cells followed by mast cell degranulation. Mast cell degranulation in turn activates mast cells to release COX-2 followed by synthesis of PGE_2_. Newly synthesized PGE_2_ induces trigeminal neurons to release CGRP and also induces TRPV1 sensitization. The influx of Ca^2+^ via TRPV1 upregulates the level of p-ERK in the TG and causes peripheral and central hypersensitivity, which induces migraine attack and pain. p-ERK, phosphorylated extracellular signal-regulated kinase; CGRP, calcitonin gene-related peptide; COX-2, cyclooxygenase-2; TRPV1, transient receptor potential vanilloid receptor 1; TG, trigeminal ganglion; PGE_2_, prostaglandin E_2_.

The surgical procedure and lowering of the electrode into the TG did not significantly increase the expression of p-ERK, CGRP and COX-2 in the TNC, ipsilateral side of the dura mater or the TG. Following electrical stimulation, a significant increase in p-ERK, CGRP and COX-2 was observed in the TNC, ipsilateral dura mater and TG compared with the sham-surgery group (P<0.001). Pretreatment with NM (6 mg/kg/day for 7 days) resulted in a significant decrease in p-ERK, CGRP and COX-2 MOD values in the TNC, ipsilateral dura mater and TG compared with the electrically-stimulated rats (P<0.05). Pretreatment with NM also demonstrated a significant increase in p-ERK, CGRP and COX-2 in the TNC, ipsilateral dura mater and TG compared with the sham-surgery group (P<0.001) and blank control group (P<0.001). No differences were detected between the sham-surgery and the blank control (Fig. 5). A schematic diagram shows the connections between p-ERK, CGRP and COX-2 in the pathophysiological mechanisms of migraine (Fig. 6).

## Discussion

The present study demonstrated that infusion of NTG and ESTG upregulated p-ERK, CGRP and COX-2 protein expression within the dura mater, TG and TNC of rats. A temporal profile of NTG-induced p-ERK was observed in the TVS. NM, a selective COX-2 inhibitor, attenuated the expression of p-ERK, CGRP and COX-2 following ESTG in rats.

Numerous hypotheses regarding the pathophysiology of migraine exist. The generally accepted neurovascular theory states that migraines are mediated by prolonged activation of meningeal nociceptors, which are located in the dura mater and vessels (1,23). The neurovascular theory centers on the activation of the TVS. The TVS consists of pseudounipolar neurons in the TG that has first-order afferent neurons innervating the pial and dural meningeal vessels, and efferent projections synapsing with second-order neurons in the TNC, which provides projections to several higher brain centers, including the posterior thalamus, hypothalamus and cortex (2,23). Activation of perivascular trigeminal nerves within meninges causes the release of CGRP, substance P and neurokinin A, which leads to a series of peripheral and central events, including inflammation and peripheral/central sensitization (24).

Central sensitization is the process that underlies migraine-associated allodynia (25). Allodynia is a state in which trigeminal neurons are elicited by persistent pain through activation of meningeal perivascular pain fibers and second-order brainstem trigeminal neurons (5,25). Consequently, meningeal perivascular pain fibers become hyper-responsive to all subsequent stimuli delivered to the receptive fields of neurons (2,6). Cutaneous allodynia, which has been found to be more common in chronic migraineurs, reinforces the hypothesis stating the necessity of frequent stimulation of central nuclei of the pain pathway to induce sensitization (7). Based on clinical symptoms, the pathophysiology of migraine can be divided into three phases: The trigger phase characterized by neuronal hyperexcitability, the aura phase involving cortical spreading depression (CSD) and the headache phase precipitated by activation and sensitization of the TVS (26,27). Central sensitization is important in the headache stages of migraine attacks and introduces the brain into a state of excessive sensitivity (2).

In rats, administration of NTG activates second-order nociceptors in the TNC and produces an increased level of nitric oxide synthase in the area associated with the central sensitization phenomenon-hyperalgesia/cutaneous allodynia (22,28). The NTG dose (10 mg/kg, for five continuous weeks, s.c.) selected for the present study was used to build a migraine hyperalgesia model that mimics chronic migraineurs. The model has provided interesting insights into the neuropharmacological mechanisms of the initiation and recurrence of migraine attacks (21,29). Thus, the present study reported a significant increase in p-ERK, CGRP and COX-2 expression in the dura mater, TG and TNC, which are the three key structures in TVS for migraine genesis following NTG administration. These results suggest that the activation of p-ERK, CGRP and COX-2 is crucial in neurogenic inflammation and central sensitization of migraine.

In addition, an interesting time-dependent mechanism for p-ERK synthesis resulting from NTG infusion was demonstrated. Numerous studies have demonstrated that the nociceptive stimulation of peripheral C-fibers could induce p-ERK in the dorsal root ganglion (DRG) (9,30). The induction p-ERK may be associated with the hypersensitivity of spinal neurons in inflammatory pain. Dai *et al* observed a transient upregulation of p-ERK minutes following TRPV1 stimulation in the DRG. However, the levels of p-ERK returned to baseline after 120 min (30). The present study demonstrated a similar time course in terms of the upregulation of p-ERK. The present study also found a transient upregulation of p-ERK in the dura mater, TG and TNC, 30 min after NTG infusion. The levels of p-ERK then gradually decreased to baseline levels before 180 min. According to our experimental observations, the NTG-induced phenomenon in rats that mimic migraine attack in migraineurs, which are characterized by continuous head scratching and climbing behavior, did not appear until 20–30 min after NTG administration. Based on the three phases of migraine pathophysiology, it was hypothesized that p-ERK primarily functions during the early onset of a migraine attack, which involves neuronal hyperexcitability and CSD. A clear time course expression of CGRP and COX-2 was not observed in TVS, suggesting that CGRP and COX-2 function in central sensitization during the headache phase of migraine pathophysiology, which maintained a migraine attack for a relatively long time period in the NTG infusion model.

ESTG, resulting in the release of CGRP, which was involved in inflammation and nociceptive information transmission, has been used to mimic neurogenic inflammation and examine migraine pathophysiology (27). ESTG has a direct effect on first-order sensory neurons, thereby causing alterations in the peripheral endings to release mediators from perivascular trigeminal nerves within meninges, which results in neurogenic inflammation (3). In the central endings, a marked activation of second-order neurons in the TNC is observed. In the present study, the ESTG models were used for a more in-depth examination of the possible functional connections between p-ERK, CGRP and COX-2 in migraine mechanisms. A significant increase in p-ERK, CGRP and COX-2 was found in the dura mater, TG and TNC of ESTG-induced rats, suggesting that the nociceptive stimulation in the TG activated the synthesis of p-ERK, CGRP and COX-2 in peripheral and central areas of the TVS, which are important in neurogenic inflammation and central sensitization during migraine. NM attenuated the expression of ESTG-induced p-ERK and CGRP in rat TVS structures. COX-2 may have stimulated the production of p-ERK and CGRP. The synthesis of p-ERK and CGRP was inhibited by the COX-2 inhibitor. Our findings are in agreement with the results obtained by Neeb *et al* who demonstrated that the activation of neuronal cells in the TG by interleukin-1β can lead to an elevated expression of COX-2 and that newly synthesized PGE_2_ (by COX-2) activates trigeminal neurons to release CGRP (16). Findings from this study support the assumption that a sequential link between COX-2 and CGRP exists. The present study also observed a downregulated expression of p-ERK in the TVS of NM-induced ESTG rats. Iwashita *et al* indicated that CSD can activate dural TRPV1 to send nociceptive signals to the TVS by facilitating degranulation of mast cells in the dura mater (11). PGE_2_, serotonin and histamine, released by mast cells, are known to induce TRPV1 sensitization (11,31). The influx of Ca^2+^ via TRPV1 upregulated the level of p-ERK and caused peripheral hypersensitivity via transcriptional regulation (32). Thus, COX-2, which synthesizes PGE_2_, can increase the synthesis of p-ERK by inducing TRPV1 sensitization. COX-2 can also transmit nociceptive signals to the peripheral and central area (11).

Thus, p-ERK, CGRP and COX-2 may function in neurogenic inflammation and central sensitization, which are relevant in migraine modulation. It was also found that p-ERK may be involved in the pathogenesis of an early onset migraine attack. In addition, the attenuation of p-ERK and CGRP release could contribute to the effect of COX-2 inhibitors, which hinder sensitization and alleviate pain. CGRP and p-ERK may improve our understanding of the mechanisms of COX-2 inhibitors in migraine therapy.
